# Anti-Angiogenesis Effect of Biogenic Silver Nanoparticles Synthesized Using *Saliva officinalis* on Chick Chorioalantoic Membrane (CAM)

**DOI:** 10.3390/molecules190913498

**Published:** 2014-09-01

**Authors:** Javad Baharara, Farideh Namvar, Marzieh Mousavi, Tayebe Ramezani, Rosfarizan Mohamad

**Affiliations:** 1 Research Center for Animal Development Applied Biology, Mashhad Branch, Islamic Azad University, Mashhad 917568, Iran; E-Mails: baharara@yahoo.com (J.B.); m_moosavi_k@yahoo.com (M.M.); 2 Department of Biology, Mashhad Branch, Islamic Azad University, Mashhad 917568, Iran; 3 Institute of Tropical Forestry and Forest Products (INTROP), Universiti Putra Malaysia, UPM Serdang, Selangor 43400, Malaysia; E-Mail: farizanmohd@gmail.com; 4 Faculty of Biological Sciences, Kharazmi University, Tehran 14911, Iran; E-Mail: tayeberamezani@gmail.com; 5 Department of Bioprocess Technology, Faculty of Biotechnology and Biomolecular Sciences, Universiti Putra Malaysia, UPM Serdang, Selangor 43400, Malaysia

**Keywords:** angiogenesis, AgNPs, chorioalantoic membrane, *Saliva officinalis*

## Abstract

Angiogenesis, which is required for physiological events, plays a crucial role in several pathological conditions, such as tumor growth and metastasis. The use of plant extracts is a cost effective and eco-friendly way to synthesize nanoparticles. In the present study, we investigated the anti-angiogenesis properties of silver nanoparticles synthesized using *Saliva officinalis* extract on chick chorioalantoic membrane. The production of nanoparticles was confirmed by the color change from yellow to brown observed after approximately 3 h at 37 °C. Then, the nanoparticles were characterized by UV-visible spectroscopy, FTIR, and TEM. The UV-visible spectroscopy results showed that the surface plasmon resonance band for AgNPs was around 430 nm. The intensity of the AgNP-specific absorption peak improved with an increase of 0.5 mL of extract into 10 mL of AgNO_3_ (2.5 mM). The FTIR results showed good interaction between the plant extracts and AgNPs. The TEM images of the samples revealed that the NPs varied in morphology and size from 1 to 40 nm; the average was recorded at 16.5 ± 1.2 nm. Forty Ross fertilized eggs were divided into four groups; the control and three experimental groups. On the 8th day, gelatin sponges containing albumin were placed on the chorioalantoic membrane and soaked with different concentrations of NPs. On the 12th day, all the cases were photographed using a photostereomicroscope. The number and the lengths of the vessels were measured using Image J software. The crown rump (CR) and weight of the embryo were also recorded. Then the hemoglobin content was measured using Drabkin’s reagent kit for quantification of the blood vessel formation. According to the data analysis, the number and length of the blood vessels, as well as the CR and weight of the embryos reduced significantly compared to the control (*p* < 0.05), dose dependently. The total hemoglobin was quantified as an indicator of the blood vessel formation. The hemoglobin content in the treated samples with AgNPs decreased, which showed its inhibitory effect on angiogenesis.

## 1. Introduction

The definition of tumor angiogenesis has been linked with Folkman’s hypothesis about the growth of solid tumors as a result of blood vessel development [1]. Angiogenesis, which is the formation of new blood vessels from pre-existing ones, is regulated by the balance of many stimulating and inhibiting factors. While physiological angiogenesis is under strike control, the disruption of this control causes the proliferation of a network of blood vessels penetrating into cancerous growth [2]. Sage belongs to the genus *Salvia* of the Labiaceae family, which comprises about 900 plant species [3]. The plant is reported to have multiple pharmacological effects, including antibacterial, antiviral, anti-inflammatory, fungistatic, antimutagenic, anticancer and antioxidative effects [4,5,6]. The leaves of *Salvia officinalis* possess some therapeutic effects due to the presence of mainly flavonoids; phenolic compounds such as carnosic, rosmarinic, caffeic and salvianolic acids and other phenolic structure-based compounds that are especially found in the alcohol-soluble fraction [7]. The application of nanoparticle materials is an emerging area of nanotechnology [8]. Metals, such as platinum, silver and gold are commonly used for the synthesis of nanoparticles by chemical and biological methods [9]. However, most of the synthetic methods reported to date rely on organic solvents, such as N,N-dimethylformamide and toxic reducing agents like sodium borohydride, which result in serious environmental issues for industrial production [10]. The green synthesis of nanoparticles offers numerous benefits of eco-friendliness and compatibility for pharmaceutical and biomedical applications as they do not use toxic chemicals in the synthesis protocols [11,12,13]. AgNPs have obvious therapeutic potential in treating a variety of diseases, including retinal neovascularization, and acquired immunodeficiency syndrome [14]. Recently, there has been increased study on anti-angiogenesis and the anti-cancer effects of silver nanoparticles [15]. These findings confirm the antitumor properties of AgNPs, and indicate that these might be a cost-effective approach for the treatment of cancer cells [16]. Since there are no reports on the use of *Saliva officinalis* to synthesize AgNPs, and, also, in respect of the antioxidant compounds and widespread growth of *Saliva officinalis*, this plant may be considered as a potential agent for the eco-friendly synthesis of silver nanoparticles. Thus, in the present study, we decided to synthesize green AgNPs using *Saliva officinalis* extract and characterize it, to investigate its anti-angiogenesis properties.

## 2. Results and Discussion

### 2.1. Synthesis of Ag-Nanoparticles and Characterized

**Figure 1:**
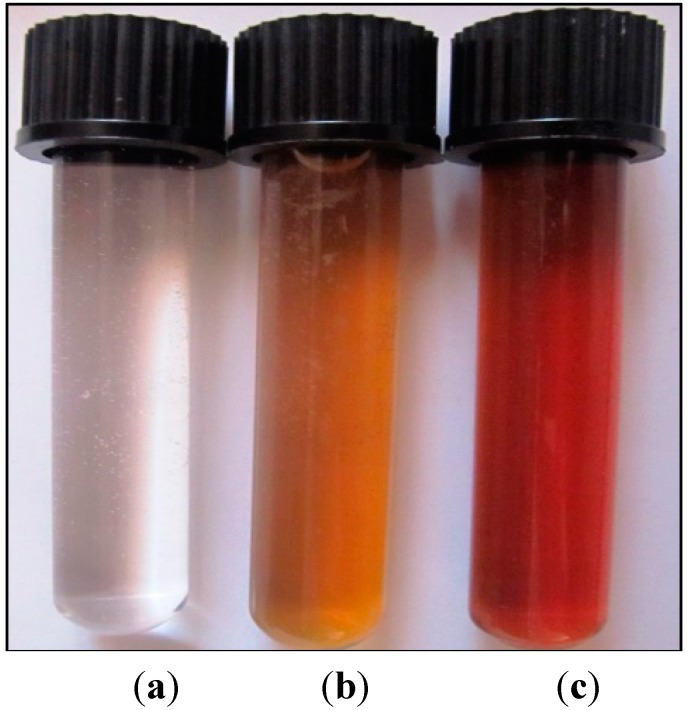
Visual appearance of vials containing the *S. Officinalis* extract and AgNO_3_ solution after different reaction times: (**a**) AgNO_3_ 5 mM, (**b**) Aerial part extract *S.* *officinalis*, (**c**) AgNO_3_.

**Figure 2:**
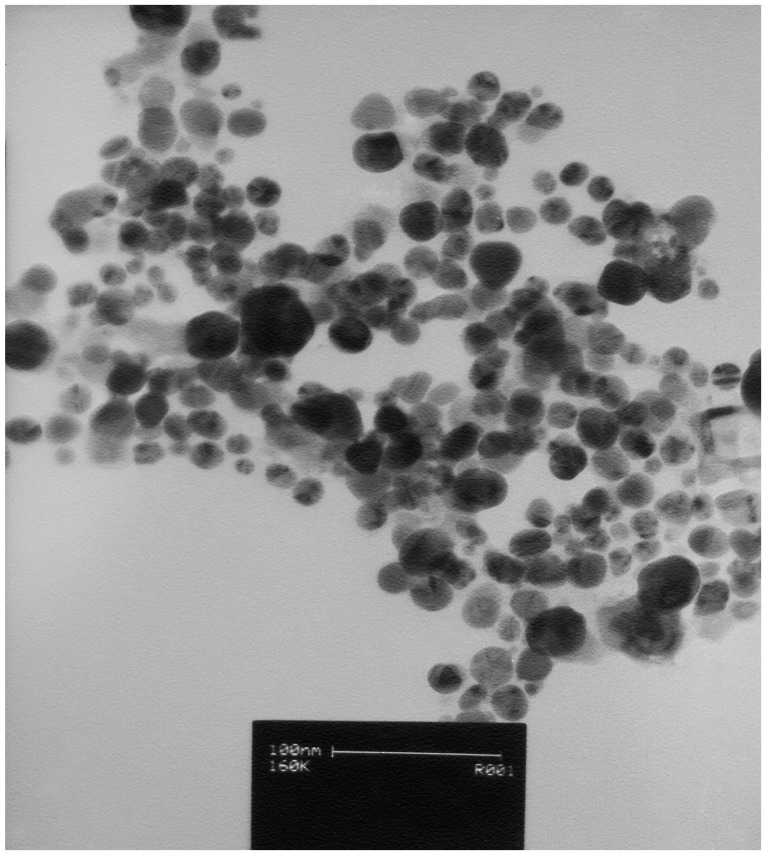
Transmission electron microscope images of the green synthesized silver NPs.

AgNO_3_ solution without the aerial plant part extract and the extract without AgNO_3_ did not show any change in color (Figure 1). Figure 2 provides an overview of the size range and distribution of the AgNPs, and shows spherical and pentagonal shapes with a size range of 1 to 40 nm, with the average being recorded at 16.5 ± 1.2 nm. From this figure, it is clear that the frequency peak comes at approximately 10–20 nm, and particle sizes ranging from 1 to 30 nm accounts for about 95% of the total particles observed.

**Figure 3:**
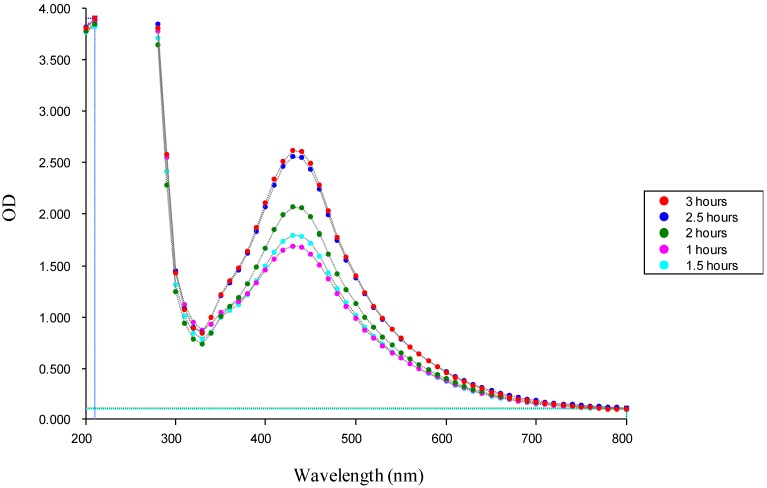
UV spectrophotometry of the green synthesized silver NPs.

**Figure 4:**
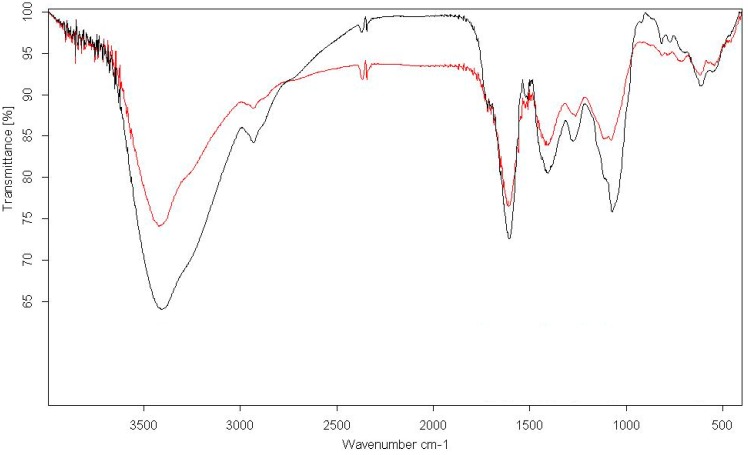
FTIR spectra of *Saliva officinalis* extract.

The absorption spectrum of the yellowish-brown silver nanoparticle solution prepared with the proposed method showed a surface plasmon absorption band with a maximum of 430 nm (Figure 3), indicating the presence of spherical Ag nanoparticles. FTIR spectroscopy was used to identify the functional groups of the active components based on the peak value in the region of infrared radiation (Figure 4).

**Figure 5:**
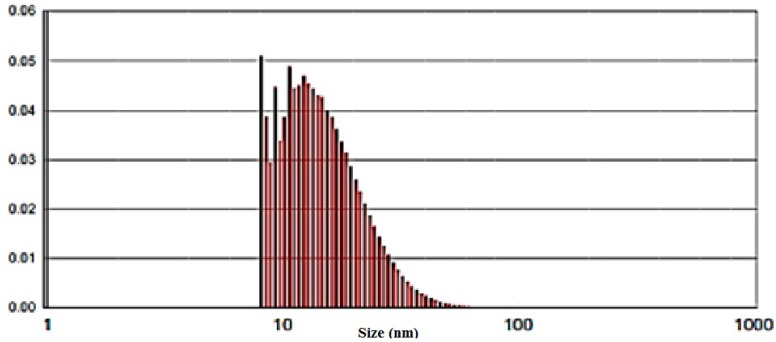
The particle size distribution of the synthesized silver nanoparticles.

The DLS results revealed that the average size of the silver nanoparticles was 21 nm. The presence of silver nanoparticles was further confirmed using EDX spectrometry, which confirmed the presence of silver. Metallic silver nanocrystals generally show a typical absorption peak at approximately 2.523 keV due to surface plasmon resonance, which increases the confidence that silver has been correctly identified (Figure 5).

### 2.2. Anti-Angiogenic Effect

**Figure 6:**
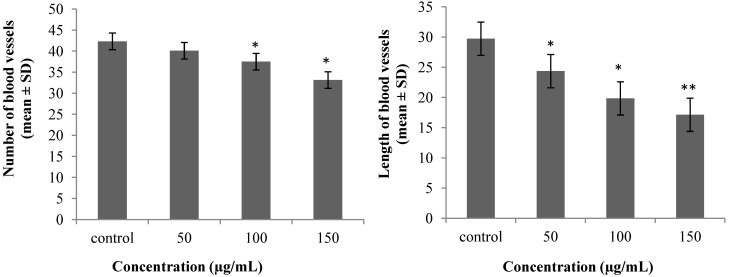
Average of number and length of blood vessels (* *p* < 0.05, ** *p* < 0.001).

The chorioallantoic membrane (CAM) assay has been proved as a reliable *in vivo* model to study angiogenesis and many inhibitors and stimulators of angiogenesis have been examined by this common method [17]. Comparing the mean of the number (42.32 ± 1.2) and length (29.72 ± 1.6 mm) of the control group with the treated samples with AgNPs at a concentration of 50 µg/mL: (40.09 ± 0.7), (24.36 ± 2.3 mm); 100 µg/mL: (37.5 ± 3.1), (19.85 ± 3.6 mm) and 200 µg/mL: (33.14 ± 2.2), (17.14 ± 1.7 mm) showed a significant decrease (*p* < 0.05). According to data analysis, the samples treated with AgNPs showed a dose dependent decrease, which is shown in Figure 6.

### 2.3. Morphometric Analysis

**Figure 7:**
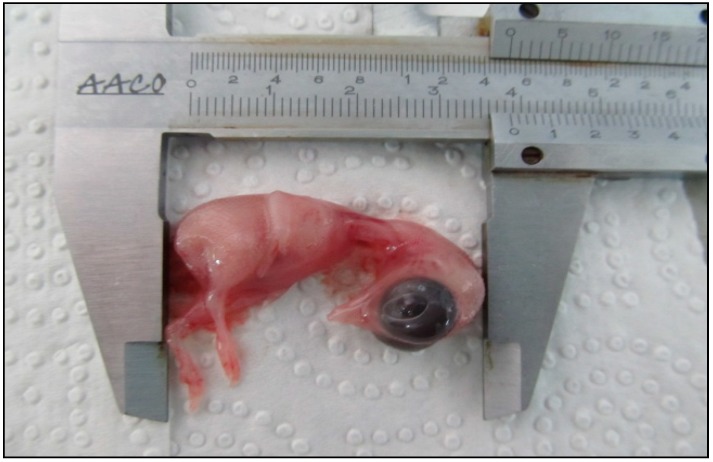
Average of CR chick embryo.

**Figure 8:**
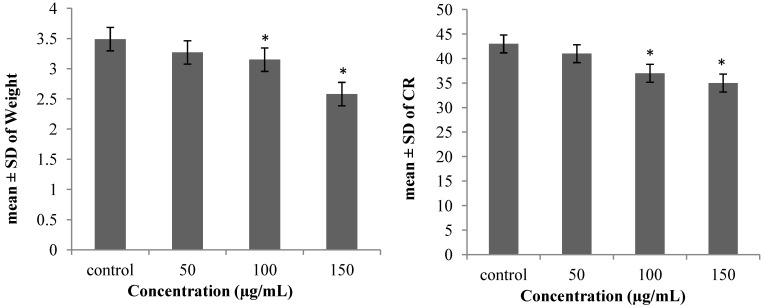
Average of CR and weight of chick embryo (* *p* < 0.05).

It is necessary to have knowledge about chick embryo development after treatment with different concentrations of AgNPs. Therefore, both the CR and weight of the treated samples were measured carefully to observe any probability of contamination (Figure 7). The CR (4.3 ± 0.2 mm) and weight (3.49 ± 0.8 g) of the control group, which were compared with the AgNPs treated samples at concentrations of 50 µg/mL: (4.1 ± 0.4 mm), (3.27 ± 0.5 g) 100 µg/mL: (3.7 ± 0.7 mm), (3.15 ± 0.1 g) and 200 µg/mL (3.5 ± 0.3 mm), (2.58 ± 0.8 g), revealed a noticeable dose dependent fall (Figure 8). Data analysis did not show any specific morphological changes, and development of the chick embryo was not affected by morphometric treatment of AgNPs.

### 2.4. Measurement of Hemoglobin

**Figure 9:**
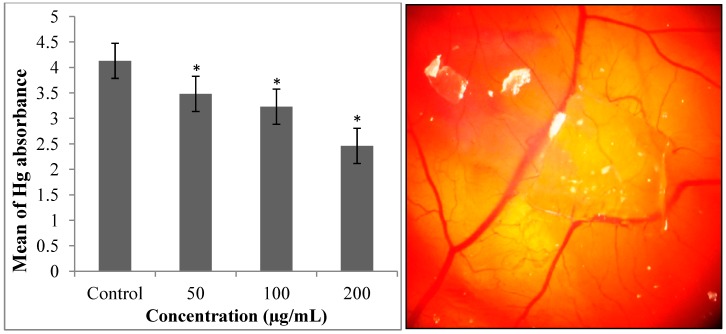
Hg absorbance (**left**) image of CAM with gelatin sponge (**right**). * *p* < 0.05, ** *p* < 0.001.

The hemoglobin was measured for the quantification of blood vessel formation, using the Drabkin method [18,19] as a measure of vessel density. This assay was used previously in other research and revealed great results that confirmed and proved the validity of the *in vivo* CAM assay [20]. The data analysis of the Drabkin test is revealed in Figure 9.

Another study also showed that silver nanoparticles led to subtle obstructive effects in the microcirculation of the chick embryo CAM [15]. These effects occurred without loss of embryo viability and were associated with the partial preservation of the capillary diameters and connectivity [21]. This particle cloud inhibited the vascular endothelial growth factor (VEGF)—induced cell proliferation, migration, and capillary-like tube formation of bovine retinal endothelial cells like PEDF (23). In addition, Ag-NPs effectively inhibited the formation of new blood micro vessels induced by VEGF. Similar studies have confirmed their inhibitory effect on vascular permeability induced by VEGF, interleukin (IL)-1β, in retinal endothelial cells [22,23].

## 3. Experimental Section

### 3.1. Reagents

Silver nitrate was purchased from Merck (Darmstadt, Germany) and used as received. Distilled deionized water was used to prepare all the needed solutions. *Saliva officinalis* were obtained from a local source (Mashhad-Iran). The fertilized eggs were purchased from Toos Company (Mashhad, Iran) and the Drabkin kit from Zistchem (Tehran, Iran).

### 3.2. Synthesis and Characterization of AgNPs

We previously reported a green method for the synthesis of silver nanoparticles using *Saliva officinalis.* Briefly, the extract was prepared by taking dried aerial plant parts (5 g) in distilled water (100 mL) and thoroughly boiling the mixture for 10 min. For the preparation of silver nanoparticles, an aliquot (0.5 mL) of the prepared extract of *Saliva officinalis* was typically added to 2.5 mM aqueous silver nitrate solution (10 mL) and kept at 37 °C until the color of the solution slowly turned from yellow to dark brown, which clearly indicated the reduction of Ag^+^ ions and formation of nanoparticles. The synthesis of AgNPs was characterized using a transmission electron microscope (Hitachi, Tokyo, Japan), DLS (Cordovan, Vaso particle, France), FT-IR (Perkin Elmer, Walthman, MA, USA) and EDX (XL 30; Philips, Eindhoven, The Netherlands).

### 3.3. Angiogenesis Assay

Forty Ross fertilized eggs were randomly divided into four groups—the control group, and experimental groups 1, 2 and 3 (treated with concentrations of 50, 100 and 200 micrograms per milliliter AgNPs), and then incubated at 38 °C and 55%–65% humidity with automatic rotation in the incubation system. On day 2 of the incubation, a window was opened in the eggs in sterile condition, which was prepared using a laminar flow hood (Telstar , Madrid, Spain), part of the shell was removed and a small window opened, which was covered by sterile paraffin and lamellas (Iran Fara, Tehran, Iran). Then the eggs were transferred to an incubator and rotated manually twice a day for normal development of the embryos. On day 8 of incubation, a gelatin sponge containing gelatin in normal saline, and albumin with 200 µL of penicillin and streptomycin was put on the chorioalantoic membrane and the experimental groups were soaked with 10 µL of AgNPs and returned to the incubator. On the 12th day of incubation, all the cases were photographed using a research photostereomicroscope (Ziess, Munich, Germany). The variables include the number and length of blood vessels that for all the samples were measured around the gelatin sponge using the Image J software. The length and weight of the samples were recorded as morphometric traits to investigate their normal development or any disorder.

## 4. Conclusions

A critical need in the field of nanotechnology is the development of eco-friendly and reliable methods for the synthesis of nanoparticles. Here, we have reported a simple biological and low-cost approach for the preparation of stable silver nanoparticles by the reduction of silver nitrate solution with a bioreduction method using *Saliva officinalis* aqueous extract as the reducing agent. The characteristics of the obtained silver nanoparticles were studied using the UV-Vis, FTIR, EDX and TEM techniques. The results conﬁrmed the reduction of silver nitrate to silver nanoparticles with high stability and without any impurity. Comparison of the experimental results showed that the average size of the synthesized silver nanoparticles was about 16.5 nm. According to the data analysis, biologically synthesized silver nanoparticles could be of immense use in medicine and could also be considered as a promising chemotherapeutic agent in cancer treatment by exhibiting anti-angiogenic properties. The CAM structure, after treatment with toxic dosages of nanoparticles appeared to be clustered with a few cellular extensions and vessel formation, and cell-spreading patterns were restricted compared to the control groups. This could be due to disturbances in the cytoskeletal functions as a consequence of the nanoparticle treatment. Lower doses were not destroyed in vascular organization. In the present study, it was demonstrated that in the CAM model, silver nanoparticles have dose-dependent cytotoxic effects on endothelial cells and inhibited blood vessel formation.
